# Entangled in complexity: An ethnographic study of organizational adaptability and safe care transitions for patients with complex care needs

**DOI:** 10.1111/jan.16203

**Published:** 2024-04-20

**Authors:** Ann‐Therese Hedqvist, Gesa Praetorius, Mirjam Ekstedt, Catharina Lindberg

**Affiliations:** ^1^ Department of Health and Caring Sciences Linnaeus University Kalmar/Växjö Sweden; ^2^ Ambulance Service Region Kalmar County Västervik Sweden; ^3^ Swedish National Road and Transport Research Institute Linköping Sweden; ^4^ Department of Maritime Operations University of South‐Eastern Norway Norway; ^5^ Department of Learning, Informatics, Management and Ethics, LIME Karolinska Institutet Stockholm Sweden

**Keywords:** care transitions, complex care needs, Functional Resonance Analysis Method, inter‐professional collaboration, organizational adaptability, patient safety, resilience

## Abstract

**Aim:**

The aim of this study was to visualize vulnerabilities and explore the dynamics of inter‐professional collaboration and organizational adaptability in the context of care transitions for patients with complex care needs.

**Design:**

An ethnographic design using multiple convergent data collection techniques.

**Methods:**

Data collection involved document review, participant observations and interviews with healthcare and social care professionals (HSCPs). Narrative analysis was employed to construct two illustrative patient scenarios, which were then examined using the Functional Resonance Analysis Method (FRAM). Thematic analysis was subsequently applied to synthesize the findings.

**Results:**

Inconsistencies in timing and precision during care transitions pose risks for patients with complex care needs as they force healthcare systems to prioritize structural constraints over individualized care, especially during unforeseen events outside regular hours. Such systemic inflexibility can compromise patient safety, increase the workload for HSCPs and strain resources. Organizational adaptability is crucial to managing the inherent variability of patient needs. Our proposed ‘safe care transition pathway’ addresses these issues, providing proactive strategies such as sharing knowledge and increasing patient participation, and strengthening the capacity of professionals to meet dynamic care needs, promoting safer care transitions.

**Conclusion:**

To promote patient safety in care transitions, strategies must go beyond inter‐professional collaboration, incorporating adaptability and flexible resource planning. The implementation of standardized safe care transition pathways, coupled with the active participation of patients and families, is crucial. These measures aim to create a resilient, person‐centred approach that may effectively manage the complexities in care transitions.

**Implications:**

The recommendations of this study span the spectrum from policy‐level changes aimed at strategic resource allocation and fostering inter‐professional collaboration to practical measures like effective communication, information technology integration, patient participation and family involvement. Together, the recommendations offer a holistic approach to enhance care transitions and, ultimately, patient outcomes.

**Reporting Method:**

Findings are reported per the Consolidated Criteria for Reporting Qualitative research (COREQ).

**Patient or Public Contribution:**

No patient or public contribution.

## 1 INTRODUCTION

Globally, millions of people live with multiple chronic illnesses, functional and cognitive impairments, mental health challenges and social vulnerability, resulting in complex care needs (Barnett et al., 2012; Chien, 2012). People with complex care needs are strongly affected by fragmented care (Leutz, 2005; World Health Organization, 2016). They are vulnerable in care transitions, in particular, immediately after hospital discharge (Glans et al., 2020). Furthermore, the single‐disease healthcare model with episodic treatment of acute illness events—which most healthcare systems are based on—may be inappropriate for people with multiple chronic illnesses or complex care needs. These patients need a broader coordination of healthcare services (Leutz, 2005). The management of complex care needs involves intricate interdependencies that increase overall complexity. Hospital discharge processes exemplify this, requiring precise coordination and effective information sharing.

Current healthcare policies that favour shorter hospital stays and increased home‐based care can inadvertently result in patients and caregivers being ill‐prepared for the transition for hospital to home (Hedqvist et al., 2020; Markiewicz et al., 2020; Vos et al., 2018). As the number of patients with complex care needs rises, care transitions create a complex web of necessary coordination and collaboration. The evolving healthcare landscape calls for organizational resilience and adaptability to protect patient safety. The research unfolds at the intersection of the care transition, where numerous complexities and organizational structures meet in collaborations. Building on our previously published Functional Resonance Analysis Method (FRAM) model of the hospital discharge process (Hedqvist et al., 2023), this study extends our understanding by incorporating patient scenarios, embodying patients' real‐world care trajectories.

## 2 BACKGROUND

### 2.1 Care transitions for patients with complex care needs

Today's healthcare system is a complex and ever‐evolving landscape, often described as a high‐risk socio‐technical system (Rasmussen, 1997). In such systems, safety performance emerges from intricate interactions and relationships (Dekker et al., 2011) like interconnected threads in a patient's care trajectory. Hospital discharge and care transitions are examples of complex processes where different care providers work together with a patient and their family to ensure a safe and smooth transition (Buikstra et al., 2020; O'Hara et al., 2020). In this context, teamwork and collaboration (Cronenwett et al., 2007), which are among the core competencies of registered nurses, become integral. The ability of healthcare and social care professionals (HSCPs) to collaborate effectively, both within and across teams, is crucial. Promoting open communication and mutual respect is key to ensuring the comprehensive well‐being of patients. Multidisciplinary team collaboration lays the foundation for effective discharge planning and care transitions. Nevertheless, we must not forget the patients and their perspective, at the very heart of this complexity.

For patients with complex care needs, transitions across care providers tend to have a critical impact on safety. When transitioning from hospital to home, disruptions due to shifts in location, care providers and level of care can result in adverse events, readmissions and even fatalities (Xiao et al., 2022). Prior research on patients with multiple chronic conditions reveals that they often encounter informational gaps and poor communication during care transitions, leading to a sense of uncertainty (Foo et al., 2023). Strengthening the ability to bridge gaps enhances patient safety (Cook et al., 2000). The possibility of successfully bridging these gaps depends on both access to information and the active participation of team members (Hedqvist et al., 2023).

Patients with complex care needs are dependent on coordination and inter‐professional collaboration during care transitions to experience cohesion in and integration of the different parts of their care. However, coordinating care and fostering collaboration across care providers remain challenging. In countries with decentralized and government‐funded healthcare systems, different authorities oversee different aspects of care, leading to potential barriers in inter‐professional collaboration due to organizational and legal constraints (Anell et al., 2012; Boyle, 2011; Danhieux et al., 2021). Furthermore, recent healthcare reforms have induced a shift from in‐hospital care to home‐based care, leading to a reduction in hospital beds and shorter hospital stays (OECD, 2023). Heavy patient caseloads for both physicians and registered nurses can result in rushed and incomplete discharge planning. This, in turn, may leave patients and their carers ill‐prepared for the transition back home, making them feel excluded from the decision‐making process (Hedqvist et al., 2020; Markiewicz et al., 2020).

### 2.2 Patient safety, resilience and organizational adaptability

In healthcare research, resilience is defined broader than merely withstanding challenges; it involves the capacity to adapt to both expected and unexpected events in a complex environment, while delivering high‐quality care (Wiig et al., 2020). Resilience focuses on how healthcare teams effectively manage variations in performance. It means not simply preventing problems, but also learning and improving from both successes and failures. Resilient health care thus provides a complementary perspective on safety (Anderson et al., 2020), contrasting with the long‐prevailing single‐factor explanations deriving from the assumption that systems are linear and causal relationships can be identified (Reason, 2000).

Health care is recognized as a complex system characterized by non‐linearity and interdependencies, where even small changes or variations can lead to unexpected and disproportionately large impacts on patient safety or system efficiency (Dekker et al., 2011). Interdependency within complex systems can be understood as the intricate and often subtle connections and interactions between various elements, including patients, healthcare professionals, clinical or administrative processes and policies. With regard to hospital discharge, this may imply that even minor variations in timing—whether an event is premature, delayed or omitted—or in precision can significantly disrupt the care continuum (Hedqvist et al., 2023). Thus, holistic and integrated care approaches are essential for safe care transitions. Moreover, adaptability is needed to manage interdependencies in complex systems. Patient safety and safety performance depend on organizational adaptability, as HSCPs adjust their strategies to respond to shifting demands. Organizational adaptability is the ability of an organization and its members to effectively navigate change and uncertainty. This involves adjusting approaches in response to environmental constraints and evolving needs (Burke et al., 2006). Such adaptability is vital for delivering high‐quality care (Anderson et al., 2020).

Optimal patient outcomes hinge on effective care transition processes, with contemporary research highlighting three critical components: knowing the patient, building strong relationships across care providers and bridging systemic gaps (Baxter et al., 2020). Consequently, care transitions that integrate all these elements are characterized as both safer and more effective. Our prior research has illuminated significant systemic gaps and identified deficiencies in collaborative team efforts that can lead to compromised patient safety during such transitions (Hedqvist et al., 2023).

Through a focused examination of inter‐professional collaboration and organizational adaptability, we aspire to identify and formulate strategies that not only address these gaps but also promote safer, more seamless and person‐centred transitions in care. By doing so, we endeavour to contribute to the body of knowledge with actionable insights that pave the way for improvements in care coordination and patient safety.

## 3 THE STUDY

### 3.1 Aim

The aim of this study was to visualize vulnerabilities and explore the dynamics of inter‐professional collaboration and organizational adaptability in the context of care transitions for patients with complex care needs.

The research questions guiding this study were: First, how do timing and precision impact patient safety during care transitions? Second, which collaborative strategies can healthcare professionals employ to increase adaptability, manage vulnerabilities and reduce risks in care transitions, particularly for patients with complex care needs?

## 4 METHODS

### 4.1 Design

**FIGURE 1:**
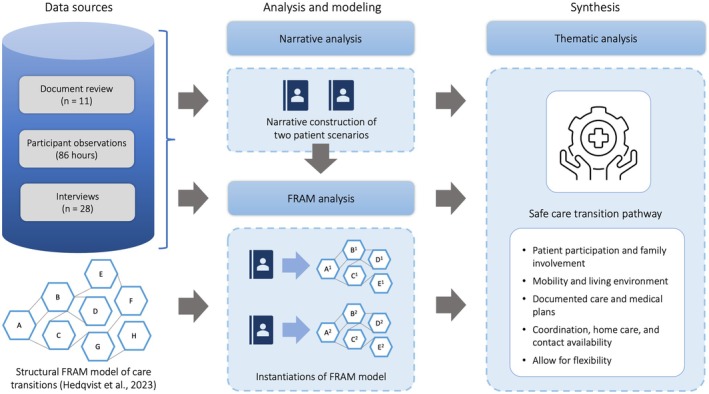
Study design, highlighting the data sources enriched by a structural Functional Resonance Analysis Method (FRAM) model (Hedqvist et al., 2023). The design outlines the subsequent steps of analysis and modelling, culminating in the synthesis of a ‘safe care transition pathway’.

This study employed an ethnographic approach (Creswell & Poth, 2017) using multiple convergent data collection techniques (Figure 1). Study designs like ethnography have been shown to be valuable in understanding the organization of health care (Sutton et al., 2016) and generating comprehensive descriptions of observations from clinical care processes (Savage, 2000). The study focused on the intricate dynamics of inter‐professional interactions across healthcare boundaries, and the organizational strategies and adaptations employed during care transitions. In pursuit of a deeper comprehension of inter‐professional collaboration and organizational adaptability during care transitions for patients with complex care needs, we immersed ourselves within multiple healthcare settings. Given the simultaneous nature of multidisciplinary activities during care transitions, traditional sequential analysis methods were considered inadequate. We utilized the FRAM (Hollnagel et al., 2014) as it offers a way to visualize and create a comprehensive description of the complexities and interdependencies in a system. Two patient scenarios were derived from the clinical context by combining insights from participant observations and interviews with healthcare professionals. The scenarios embody the real‐world intricacies of the findings. The patient scenarios complemented the organizational perspective on inter‐professional collaboration in care transitions, promoting an understanding of the patient care trajectory. Thematic analysis (Braun & Clarke, 2006) was applied to synthesize the findings and establish a safe care transition pathway.

The Consolidated Criteria for Reporting Qualitative research (COREQ) were used to report on this study (Tong et al., 2008; Appendix S1).

### 4.2 Study setting

Sweden has a decentralized healthcare system. Regions oversee primary to specialist care and municipalities are responsible for community health care and social care, encompassing services such as elderly care and nursing homes (https://www.socialstyrelsen.se/en/about‐us/healthcare‐for‐visitors‐to‐sweden/about‐the‐swedish‐healthcare‐system/, Anell et al., 2012; Swedish Healthcare System, 2020). Healthcare and social care sectors operate under distinct governance systems: the Health and Medical Services Act and the Social Services Act respectively (SFS, 2001, 2017). Although the national government provides overarching healthcare directives, the autonomy of regions and municipalities enables customization of services to meet the specific requirements of local citizens. Such self‐governance not only encourages adaptability but can also lead to fragmented and inconsistent healthcare standards across regions. Currently, approximately 36% of the population in Sweden aged 80 years or older needs support through home care, home services or nursing home residency. The number of individuals in this age group accessing community home care has seen a significant increase, doubling over the course of 8 years to 191,000 enrolees in 2018 (National Board of Health and Welfare: Vård och omsorg om äldre. Lägesrapport, 2020). This highlights the extensive healthcare demands of this demographic and indicates a shift towards more community‐based care delivered in patients' homes.

The region selected for this study is characterized by a large older population. With its diverse mix of urban, rural and sparsely populated areas, the region's unique characteristics reflect the inherent challenges and opportunities of a decentralized healthcare structure. Given its robust regional and municipal collaborations, this region offers a suitable perspective for examining complex healthcare dynamics.

### 4.3 Recruitment of participants

We employed purposive sampling to gather a broad spectrum of data from HSCPs involved in care transitions. This approach aimed to include participants in diverse roles and fields, integral to managing care transitions of patients with complex care needs. The process began with project briefings to operational managers within each organization, using both written and verbal communications. After gaining consent, we approached department heads to secure access to their teams. All but one department agreed, with the pandemic's impact cited as the reason for non‐participation. Department heads then relayed the study details to their staff, who were made aware of their right of voluntary participation and subsequently gave informed consent prior to their involvement in observations and interviews.

The sample consisted of 37 HSCPs from various domains, including in‐hospital care, ambulance care, primary care and community care. Our inclusion criteria specifically targeted professionals who had direct involvement in coordinating, executing or facilitating transitions of care. We excluded professionals who, although integral to the broader healthcare team, were not directly involved in transition processes. This ensured that the perspectives of our participants remained focused and pertinent. This diverse group of participants encompassed a wide range of professions and roles, such as registered nurses, physicians, ambulance nurses, occupational therapists, physiotherapists, social service officers, care coordinators and assistant nurses. The group composition provided a multidisciplinary and comprehensive perspective of HSCPs engaged in delivering health care and social care during care transitions, as illustrated in Table 1.

**TABLE 1:** Overview of participants in the study.

		Interviews		Participant observations	
		Informal interviews (*n* = 15)	Formal interviews (*n* = 13)	Individual observations (*n* = 11)	Meeting observations (*n* = 5)
	*n*	*n*	*n*	*n*	*n*
Medical or geriatric hospital ward					
Registered nurse	5	4		4	3
Care coordinator	4	3	1	2	4
Physician	3	3			1
Ambulance care					
Ambulance nurse	3		3		
Primary care					
Care coordinator	4	2		2	4
Physician	1		1		
Community care					
Occupational therapist	2		1		2
Physiotherapist	3		1		3
Registered nurse	2		2		2
Care coordinator	3	3		3	3
Social service officer	4		1		4
Assistant nurse	3		3		
Participants total	37	15	13	11	26

### 4.4 Data collection

From June 2020 to October 2021, we utilized a variety of complementary data sources. Document reviews gave us an understanding of the formalized structures in the healthcare system. Through participant observations of individual HSCPs and inter‐professional meetings, we were able to observe real‐time collaborations and adaptability. Interviews with HSCPs revealed the motivations, challenges and perspectives that influenced behaviours and actions performed during transition processes.

#### 4.4.1 Document review

In the summer of 2020, we began collecting documents for the review. Documents were collected by the first author (AH) and used to formulate observation protocols and craft interview questions for subsequent interviews. We sourced key documents from government websites and reviewed commonly utilized online and physical materials across the care units we observed. This comprehensive collection encompassed 11 documents, including national laws, regional guidelines and local procedures from both hospital and community care settings. In the initial stages of the review process, the first author (AH) conducted a meticulous examination of the documents. This involved a detailed reading and thematic coding of the contents (Bowen, 2009), which allowed for a deeper understanding of the formal policies and procedures governing hospital discharges and the intricacies of inter‐professional collaboration. To ensure a comprehensive analysis, the findings from this primary examination were regularly discussed and deliberated on among all authors, facilitating a collaborative interpretation and refinement of the themes identified. This understanding informed our selection of professions to observe and pinpointed critical areas and interactions for focused attention in the study.

#### 4.4.2 Participant observations

To uncover and understand the intricacies of communication and interaction patterns during care transitions, we performed participant observations (Spradley, 2016). Through an ethnographic approach, we observed activities, behaviours and roles in interactions over an extended period to gain an in‐depth perspective of the environment from within (Creswell & Poth, 2017). We shadowed HSCPs in their daily routine activities and observed key meetings involving coordination, planning and patient discharge discussions. To capture the essence of inter‐professional collaboration and organizational adaptability in everyday work, we immersed ourselves in a variety of scenarios. The observations were conducted in multiple healthcare environments including hospital wards (17 sessions), primary care (4 sessions) and community care settings (eight sessions). The observation sessions were carefully structured and limited to no more than 3 h at a time, to maintain focus and reduce observer fatigue. In total, we conducted 11 individual observations of HSCPs and observed five inter‐professional meetings, for a total of 86 h.

Our observation protocol, shaped by insights from the document review, focused on key aspects of care transitions and hospital discharges, including coordination, communication and information sharing. Our observer (AH), a professional nurse, was recognizable to those being observed while remaining unobtrusive, fostering an environment conducive to insightful observation and, when appropriate, interaction. This approach yielded a deep understanding of practices without disrupting them (Spradley, 2016). To protect patient privacy and avoid recording sensitive information, no audio recordings were made during meetings. Instead, observations were documented using established participant observation methods, with field notes transcribed on the same day to capture details and context for analysis.

#### 4.4.3 Interviews

The first author (AH) performed interviews probing the contexts and rationales behind the actions we saw, to enrich the observational data. Informal conversational interviews with healthcare professionals across the care spectrum helped illuminate the reasons and methods behind their actions (Creswell & Poth, 2017), with a focus on adaptability and collaboration during care transitions. We conducted 15 informal interviews on‐site, concurrently with observations, taking careful notes during each discussion. To gain a deeper understanding and address the insights not captured through observations alone, we also performed 13 formal interviews with key informants. These individuals were specifically chosen for their expertise and knowledge of collaborative practices during care transitions. The interviews were conducted either face‐to‐face, by phone or via digital platforms like Skype or Zoom. The interview guide remained dynamic and evolved throughout the study, allowing for the exploration of emerging themes and the refinement of understanding based on the collected data. An example of an interview question is ‘Could you describe an incident where a patient's discharge plan didn't unfold as anticipated, and how you addressed this?’ This question sought to elicit rich narratives, offering insights into the practical implementation of policies and the adaptability of healthcare professionals. The formal interviews were recorded and transcribed verbatim for analysis.

### 4.5 Data analysis

To understand the complexities of care transitions, we performed an analysis using a multi‐layered approach. It began with a narrative analysis, to encapsulate the subtleties observed in real‐world settings. Next, the FRAM was applied to probe into the variabilities encountered in actual care scenarios. The process culminated in a thematic analysis that integrated and synthesized the findings.

#### 4.5.1 Narrative analysis

Narrative construction was utilized to develop applicable patient scenarios derived from participant observations and interviews. Guided by Polkinghorne (Polkinghorne, 1995), two narratives were crafted based on the clinical context. The narrative analysis began with ‘emplotment’, where pivotal events from data were interconnected into a unified storyline. This was followed by thematic extraction where recurring themes were identified and emphasized. Finally, temporal sequencing ensured that the narratives followed a logical, chronological order. As a result, two representative patient scenarios were constructed, referred to as John and Elsa. These are shown in Boxes 1 and 2. The scenarios played a pivotal role in the subsequent analysis, helping us weave the patient care trajectory into this work. Although the narratives of John and Elsa were constructed from real interviews and observations within clinical settings, they are composites. To ensure confidentiality, elements from multiple situations and patients were integrated into each scenario. Still, these narratives closely mirror authentic scenarios encountered or potentially seen in care transitions for patients with complex care needs.

**BOX 1:** Scenario 1—John.

*John* John, an 80‐year‐old man with worsening chronic heart conditions, was quickly discharged from the hospital on a Friday due to pressing demand for beds. His release was rushed with a hastily prepared care plan. At the hospital, he was assessed and deemed to be in his usual state, so no extra support or aids were provided for his homecoming. John, struggling to voice his needs, consented to discharge after a brief discussion with the physician. He was sent home with the understanding that home care staff would attend to him, yet the staff received scant information about his discharge, and his family was not notified. At home, John's condition had noticeably declined; he had trouble getting up from his chair and walking to the bathroom, overwhelming the home care team tasked with his care. The day after discharge, a home care assistant nurse sought advice from the community nurse due to John's worsening condition. The community nurse, unfamiliar with John's history and engaged with another patient elsewhere, lacked immediate access to his digital records. She contacted the on‐call general practitioner in primary care, who also lacked knowledge of John. With it being the weekend, John's medical summary was not yet updated in the system, leaving the physician with incomplete information for decision making. Given the circumstances and staff limitations, the physician suggested a possible hospital readmission for John if necessary. Meanwhile, the nurse, faced with a staff deficit, advised the home care team to call an ambulance for John's assessment rather than wait for a nurse's visit. Around 40 min after the call, ambulance clinicians arrived and assessed John. Observing symptoms of chronic heart failure, they sought clarity on his baseline health and post‐hospital care plans, but the home care staff were unaware of any detailed medical plans from his recent hospital stay. Knowing only that John was deemed ready for discharge, the ambulance clinicians, lacking a definitive care plan, opted to take John back to the emergency department. He was swiftly readmitted to a medical ward, treated and sent home within a day, without any apparent improvement in his condition.

**BOX 2:** Scenario 2—Elsa.

*Elsa* Elsa, an 83‐year‐old woman, was hospitalized due to a pelvic fracture from a fall and developed pneumonia during her hospital stay. After a period of treatment, a discharge planning meeting was held to arrange her home care. Typically, rehab specialists would assess a patient's mobility, but due to limited availability, this task fell on a ward nurse. The nurse's assessment was that Elsa was ready for home rehabilitation and she was subsequently discharged with an oral antibiotic treatment for pneumonia. Transport for Elsa was organized, but the lack of an elevator in her building was overlooked. Upon arrival, the assistant nurses expected Elsa to be mobile enough to manage on her own. However, on discovering that she could not climb a flight of stairs and with the taxi driver unable to assist, staff was forced to summon extra help to carry Elsa to her apartment. This resulted in an extended wait for everyone involved. Upon entering her apartment, it was clear Elsa could not use her walker effectively due to weakness from her hospital stay and the pain from her pelvic fracture. The thresholds in her home, which had not been considered during her hospital mobility training, added to her discomfort. The care staff worked to obtain a temporary wheelchair, extending their stay and foregoing breaks to accommodate her needs, ultimately impacting the day's entire care schedule for the staff and other waiting patients.

#### 4.5.2 FRAM analysis

Our analysis utilized the FRAM (Hollnagel et al., 2014) to visualize complexities and variabilities during care transitions. The FRAM is a systems thinking approach that aids visualization and analysis of interactions within socio‐technical systems. It highlights how variability affects system performance, potentially leading to unexpected or adverse outcomes (Hollnagel et al., 2014). In the FRAM, system actions, known as ‘functions’ and depicted as hexagons, are examined for their performance under different conditions. For detailed information on the FRAM and its application, Hollnagel's foundational work (Hollnagel et al., 2014) is recommended. Further insights into applying the FRAM to care transition processes can be found in Hedqvist et al. (2023).

In a FRAM analysis, two steps are crucial. The initial step involves mapping out and constructing a system model of the process, as detailed in our prior work (Hedqvist et al., 2023). This model delineated the discharge process from hospital admission through the first 72 h post‐discharge. This model represented potential or theoretical variability within the care transition process. The second step, ‘instantiation’, brought the model to life by applying it to the real‐world scenarios of John and Elsa. This step revealed the system's dynamic behaviour, illustrating how various functions interacted and influenced each other under actual, real‐life conditions (Hollnagel et al., 2014). It cast light on the actual interdependencies between patients and HSCPs, providing a detailed picture of a possible real‐life care transition.

**FIGURE 2:**
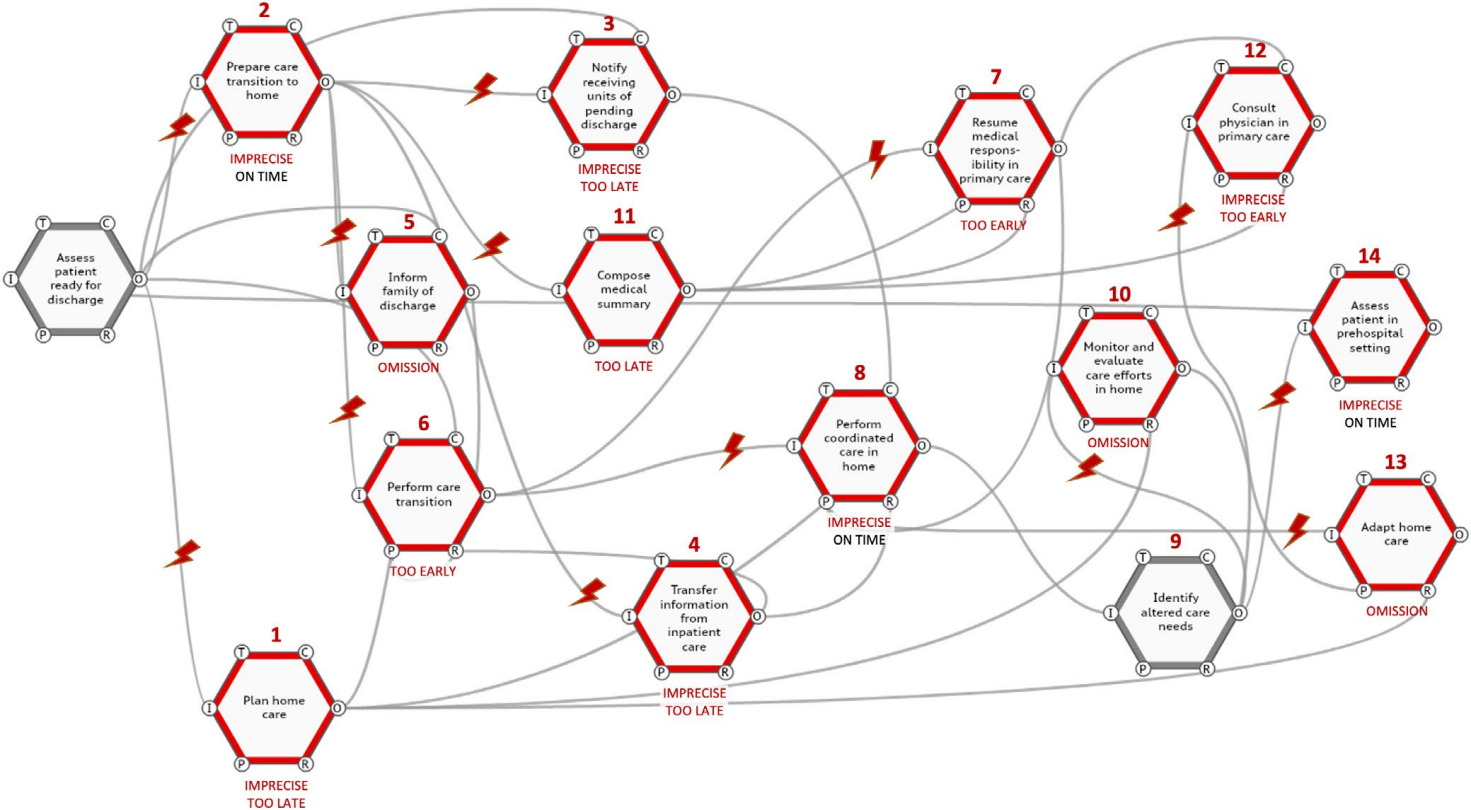
Instantiation of the Functional Resonance Analysis Method (FRAM) model for John's scenario. Red hexagons indicate functions and lightning bolts indicate points of concern resulting from issues of timing or precision in the execution of functions.

**FIGURE 3:**
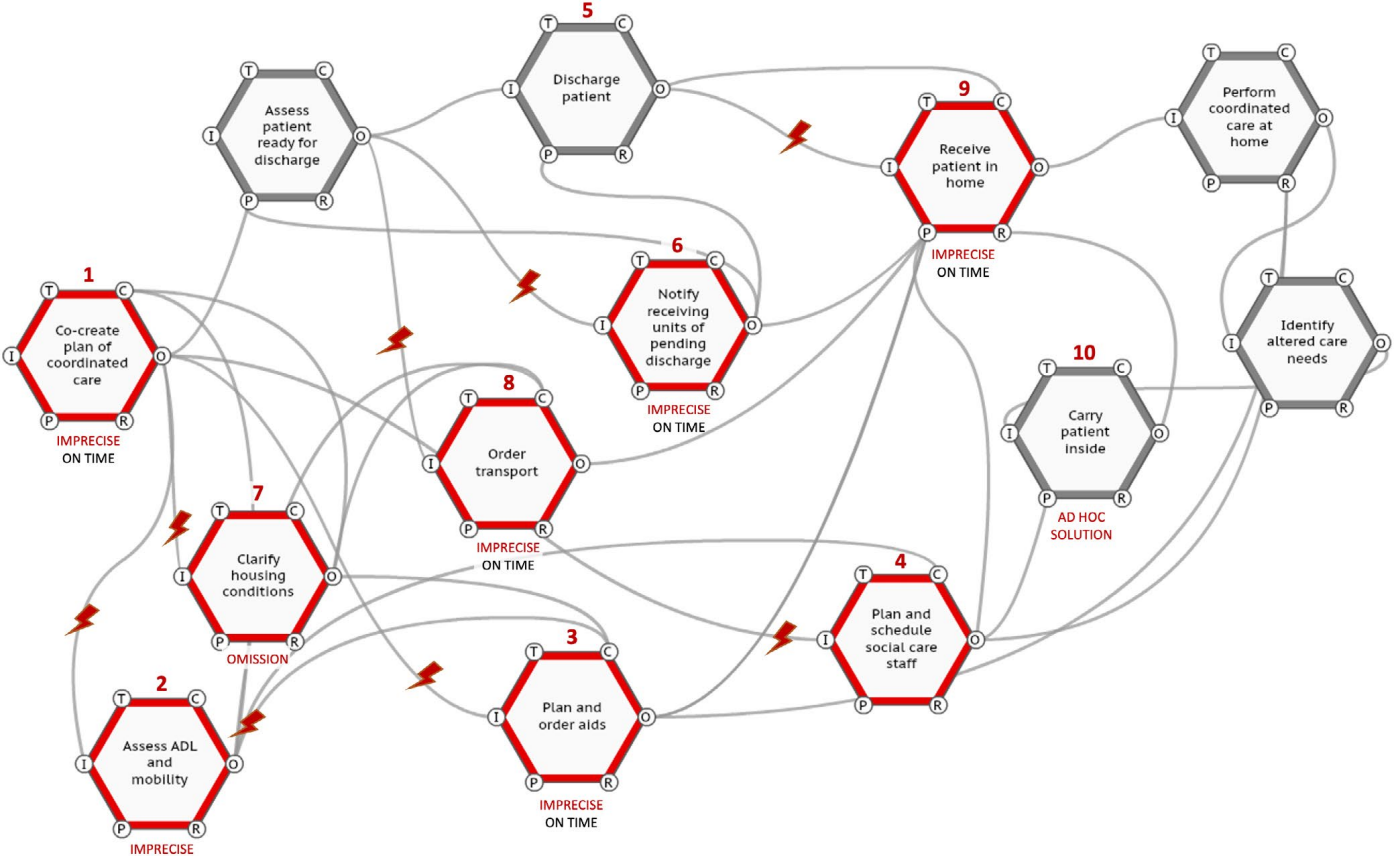
Instantiation of the Functional Resonance Analysis Method (FRAM) model for Elsa's scenario. Red hexagons indicate functions and lightning bolts indicate points of concern resulting from issues of timing or precision in the execution of functions. ADL, Activities of Daily Living.

We began by reading the constructed narratives, to elucidate what was happening during John and Elsa's scenarios. Next, we dissected the scenarios to discern the functions at play. We compared and further developed the function definitions previously framed in the FRAM model (Hedqvist et al., 2023). We then graphically depicted these functions and their interrelations for each patient scenario (see Figures 2 and 3), using the ‘FRAM Model Visualizer’ (FMV, 2016 https://functionalresonance.com/the%20fram%20model%20visualiser/) as an aid. The visual maps utilized hexagons to represent functions, and lines to illustrate their connections, highlighting the dynamics of each care scenario. Red lightning bolts were strategically placed to indicate points of variability in function execution, bringing attention to where such variations occurred within the patient trajectories. Next, the aggregation of variability was assessed, specifically focusing on instances where variability in timing and precision converged, giving rise to the potential for functional resonance. Finally, strategies for managing this variability were suggested.

#### 4.5.3 Thematic analysis

After this, a thematic analysis (Braun & Clarke, 2006), a pivotal component of ethnographic methodology (Creswell & Poth, 2017), was used to integrate and synthesize the findings from the FRAM analysis and the patient scenarios. The thematic analysis unfolded through an iterative and in‐depth process. It began with initial coding, where the collected data were dissected into meaningful units for detailed examination. Following this, we organized these initial codes into broader themes and sub‐themes, to identify patterns and capture the richness and diversity of the data. We then reviewed and refined the themes to ensure they were a true representation of the dataset as a whole. During this iterative process, we returned to the original documents, observations and interviews, to confirm and solidify the emerging findings. The final stage involved distilling the core message of each theme, giving it a descriptive label. This step was pivotal in synthesizing the nuanced lived experiences and insights into coherent, articulated themes that informed our understanding of the complex dynamics of care transitions.

During the analysis, special attention was paid to the patterns of real‐world implications emerging in the narratives. These patterns were then juxtaposed with the functional variabilities and interactions illuminated by the FRAM models, enriching our understanding of care transitions from multiple perspectives. Continuous dialogues between the authors revealed underlying patterns, providing insights into the complex dynamics of care transitions and underscoring possible implications.

The end product of this analysis was the formulation of the ‘safe care transition pathway’ (Box 3). This pathway is a reflective tool, informed by empirical data and the ethnographic principle of understanding human behaviour in its context. It encapsulates five thematic pillars that anchor the core requirements for adaptability and seamless inter‐professional collaboration. Each theme represents a critical leverage point for action, with the potential to guide healthcare professionals in adopting a proactive and resilient stance in the multifaceted challenges of care transitions.

**BOX 3:** Suggestion of a ‘safe care transition pathway’ for patients with complex care needs.

*Safe care transition pathway for patients with complex care needs*
Before discharge, ensure the following steps are undertaken
*Patient participation and family involvement*
Adopt a person‐centred approach. This emphasizes understanding and respecting the preferences and abilities of both the patient and their family Keep the patient and their family well informed about the discharge process. Ensure that they understand every step and, whenever possible, involve them actively in the planning and decision‐making processes Confirm that both the patient and their family are ready and in consensus regarding the discharge and the patient's return home
*Mobility and living environment*
Perform an Activities of Daily Living (ADL) assessment overseen by qualified rehabilitation specialists to understand and discern the patient's abilities and mobility constraints Examine the patient's living environment to ascertain its suitability and accessibility for recovery Ensure that essential aids are accessible to the patient within the initial 24 h after being discharged Ensure that the transport arranged from the hospital is tailored to the patient's actual housing conditions. Factors such as stairs, elevators and other unique features must be considered to guarantee the patient can safely enter their home Verify that the patient, given the available aids and their living environment, can perform essential activities such as resting, eating and using the toilet
*Documented care and medical plans*
Provide the home care team with a comprehensive discharge summary, detailing the patient's condition, treatment provided, ongoing care needs and any potential warning signs to watch for Establish a comprehensive care plan detailing steps for potential health deteriorations, specifying when and which healthcare level to approach For patients transitioning to palliative care or under a do‐not‐resuscitate directive, a specific medical plan should be documented and communicated to all involved parties, including the receiving units, patient and their next of kin Ensure that there is a clear protocol in place for exacerbations or emergencies, detailing steps to be taken, numbers to call and immediate actions
*Coordination, home care and contact availability*
Arrange an inter‐professional conference with home care providers before the discharge. This should involve discussions about the patient's medical needs, medication routines, necessary equipment and rehabilitation requirements Assign a care coordinator for the patient. This person will be the primary contact for the patient and home care providers, facilitating communication and addressing concerns Ensure that medications are available and organized. Supply newly introduced medications for the first days, if possible, to promote continuity of treatment Initiate a dialogue with the designated contact person within the home care service Provide the patient and their next of kin with 24/7 contact details in case of any concerns or emergencies
*Allow for flexibility*
Allocate specialized staff from home care or social services for the first few days following discharge. This team will coordinate essential health and social care actions, implement required adjustments and provide extended personal care for the patient as needed

### 4.6 Ethical considerations

The study was approved by the Swedish Ethical Review Authority (Registration number 2020‐01219). Studies utilizing participant observations not only offer valuable insights into human behaviour, but also come with ethical responsibilities. Hence, ethical rigour in participant observations is not only a moral imperative, but also crucial for the credibility and trustworthiness of scientific research, respecting the rights and dignity of the individuals being observed. Great efforts were made throughout the study to uphold the principles of informed consent, anonymity, transparency and cultural sensitivity during the data collection and to minimize potential harm. Pseudonyms were used to protect the identities of participants. Continuous reflection on the role and impact of the observer promoted sensitivity and safeguarded both the integrity of research and the well‐being of participants.

### 4.7 Trustworthiness and reflexivity

To enhance the trustworthiness of this study, a range of strategies were employed. These included member checking, peer debriefing and data triangulation (Creswell & Poth, 2017). Purposive sampling ensured that participants had relevant experiences, increasing the study's depth and relevance. Interview techniques featured open‐ended questions and active listening, encouraging candid participant narratives and capturing nuanced experiences. Reflexivity was integral, with ongoing team discussions used to maintain objectivity and self‐awareness in data interpretation.

## 5 RESULTS

The findings are presented in five sections as follows. First, patient scenarios are narrated, embodying the care transition process through the patient care trajectory. From these scenarios, functions within the care transition process are identified and outlined. Moving from the potential variability highlighted in the FRAM model, we present the actual variability in the care transition processes derived from the patient scenarios. Next, the impacts from aggregation of system variability in timing and precision are described, delineating the complexities of this system variability across different scenarios. Finally, we suggest a structured pathway for safe care transitions, containing a set of conditions to manage the aggregation of system variability and promote patient safety.

### 5.1 Patient scenarios embodying the care transition process

The narrative analysis resulted in the construction of two illustrative patient scenarios: John and Elsa. John, an 80‐year‐old man with a heart condition, experienced a hasty discharge from hospital (as detailed in Box 1). Once home, his health took a turn for the worse. Communication breakdowns and the unavailability of his medical records led to his readmission. Elsa, 83 years old, was admitted to hospital due to a pelvic fracture after a fall with ensuing pneumonia (Box 2). A premature discharge, lacking a thorough mobility evaluation, resulted in adjustments for community care that not only disrupted the care staff's routine, but also influenced care timing for other patients.

### 5.2 Functions in the care transition process

The analysis of the patient scenarios uncovered 39 distinct functions integral to the care transition process for patients with complex needs. Table 2 highlights a selection of key functions, chosen for their significant impact on patient outcomes and their strong interdependencies within the system. These interconnections underscore critical risk points. For the full scope of functions identified, including in‐depth descriptions and functional aspects, see Appendix S2.

**TABLE 2:** A selection of functions occurring in the care transition process, modelled by the FRAM.

Function	In scenario		Description
	John	Elsa	
Assess ADL and mobility		√	The ADL (Activities of Daily Living) and mobility assessment is an evaluation of a patient's capacity for self‐care and independent movement. It encompasses activities such as eating, dressing and bathing, as well as assessing balance, gait and the requirement for mobility aids. Typically conducted by rehabilitation experts such as physiotherapists or occupational therapists, the assessment informs the level of support and equipment needed after hospital discharge. If specialized rehabilitation staff are unavailable, ward nurses are responsible for this assessment. The insights gained from this process are integral to developing a coordinated care plan that facilitates a patient's seamless transition from hospital to home care
Assess patient in pre‐hospital setting	√		If there is a perceived need of pre‐hospital assessments and care, the community care staff, family or patient may call emergency services. The pre‐hospital assessment refers to the medical evaluation and care provided to a patient before arrival at hospital, typically conducted by ambulance personnel. This evaluation plays a crucial role in determining the immediate needs of a patient and formulating a preliminary treatment plan
Assess patient ready for discharge	√	√	Following medical treatment, a patient's health and medical progress are evaluated to determine their readiness for discharge. The attending physician makes a decision on this based on the assessment results. If the patient requires modifications at home, such as medical equipment, accessibility enhancements or education for family members, the discharge might be delayed. Furthermore, if there are concerns about the patient's capabilities due to medical, psychological or environmental factors, discharge may be postponed until necessary arrangements are finalized
Co‐create plan of coordinated care		√	Collaborative planning involves a multidisciplinary team, including rehabilitation experts, social service officers, physicians, nurses and other specialists, to formulate a holistic care plan. This plan considers the patient's medical needs, preferences and challenges. After thorough discussions among all parties, a preliminary care plan is co‐created to guide the patient's transition and ongoing care
Identify altered care needs	√	√	Identifying altered care needs is a critical aspect of adaptive and person‐centred health care. As a patient progresses, recovers or faces new health challenges, their care requirements can change. Through a systematic assessment of the patient's current health status in relation to their previous status, healthcare professionals aim to pinpoint these changes. Such identification ensures that the patient receives the most appropriate and effective care tailored to their evolving needs
Plan home care	√		Community home care nurses facilitate the patients' seamless transition home by addressing key care elements. They handle wound care, prepare supplies and liaise with specialists as required. Medication management involves reviewing prescriptions and ensuring accurate dosages. They also coordinate the delivery and setup of medical equipment and provide essential training. Nurses educate patients and their families for confident home care. A proactive monitoring strategy with follow‐up visits is established to refine care as needed. Collaboration with other healthcare professionals solidifies a comprehensive approach to patient well‐being
Receive patient in home		√	Upon patient discharge, home care or rehab staff are ready to receive the patient in the home. This entails healthcare professionals being informed, prepared and scheduled for the patient's needs. Community care ensures that necessary resources, like medical equipment, are available. Clear communication with social care staff is vital for understanding patient needs. Care is customized to the patient's medical history, current status and emotional well‐being. Safety is prioritized, with instant support in mobility, medication and wound care, ensuring a safe and supportive patient environment
Resume medical responsibility in primary care	√		The general practitioner in primary care receives a referral from the attending physician at hospital, providing guidelines for post‐hospitalization follow‐up or additional medical actions. This referral can also outline aspects of home‐based care

### 5.3 Actual variability in the care transition process

The application of the FRAM model to real‐world scenarios illuminated the actual variability in care transition processes. By analysing specific patient scenarios, we uncovered tangible variability affecting outcomes. We observed that both the timing of care activities—too early, on time, too late or omitted—and their precision—ranging from high or acceptable to low—significantly impacted subsequent stages in the care transition trajectory for patients like John and Elsa. These real‐world variabilities are represented in two simplified subset models of the FRAM model, focusing on key functions and their interdependencies that significantly affect patient safety. These models provide critical insights into the real‐world impact of variability in care processes.

#### 5.3.1 Scenario 1: Timing of the hospital discharge

To illustrate the timing challenges in hospital discharges, we use John's case as a prime example (see Box 1). The instantiated model for John's scenario focuses on the adaptability at an organizational system level and the specific variabilities involved. For clarity in the visual representation, we have included only the functions that are crucial to understanding this narrative (see Figure 2), with key functions in red, clearly numbered for easy reference (see Appendix S2 for a full description).

To free up hospital beds, John's discharge procedure was expedited. The following quote from a physician underscores the complex decision making involved in managing hospital discharges, where time efficiency must be carefully weighed against the patient's health and the broader implications for their continued care:

The short duration of care also means that sometimes there is a risk taken in discharging and sending the patient home. Sometimes it works out, sometimes it doesn't. It has to be a gamble. (Participant 25, physician, medical ward)

Rapid discharge processes inherently involve taking calculated risks. At times, the expedited discharge of patients is successful; at other times, it leads to adverse outcomes. In John's scenario, patient safety was jeopardized mainly by discrepancies in timing during the inter‐professional collaboration and the interaction in information dissemination. The delay in care planning (Function 1) and imprecise preparation for care transition (Function 2) led to delayed and flawed communication with the awaiting care units (Functions 3 and 4). Additionally, John's family was left uninformed (Function 5), and the transition occurred on a Saturday (Function 6). This led to restricted access to regular staff and a lack of relational continuity, increasing the risk of gaps in care. The lack of communication with John's general practitioner (Function 7) indicated that a plan for his primary care had not been established. Deficiencies in preparation and inaccurate data meant that essential requirements for John's mobility were not met, disrupting the coordinated home care (Function 8). Incomplete patient data and staff shortages impeded the recognition of John's evolving care needs (Function 9), compelling a community nurse to omit a necessary assessment of his condition (Function 10). With the general practitioner not fully briefed (Function 11) and the attending physician's decisions still pending (Function 12), no home care adjustments were made (Function 13). This left John with inadequate home care support. The information gap prompted home care staff to call for an ambulance (Function 14). The ambulance team, without access to John's medical history, opted to return him to the hospital for lack of a better solution. An ambulance nurse reflects on the predicament:

Without medical records or a care plan, I'm in the dark. All that's left is the emergency room. (Participant 30, ambulance nurse)

#### 5.3.2 Scenario 2: Precision of assessment and planning of care

Elsa's scenario, as outlined in Box 2, exemplifies the criticality of accurate assessment and care planning. Figure 3 illustrates a streamlined version of the organizational responses and particular variabilities in Elsa's situation. This model demonstrates how imprecision and inefficiency in key functions can force downstream organizations to allocate extra time and resources to a patient.

The coordinated care plan for Elsa (Function 1) was compromised by inaccuracies. A crucial error occurred in the assessment of her activities of daily living (ADL) and mobility (Function 2). Due to the perception that involvement of a rehab team was unnecessary, the assessment was not conducted by rehabilitation specialists, but by a ward nurse.

Occupational therapists like myself are trained to discern patients' behaviors and mobility. By observing them, say, sitting in a chair, we gather critical information. Ideally, occupational therapists should perform these assessments, but we aren't always called upon. (Participant 14, occupational therapist, community care)

This lapse led to further issues with arranging the proper aids (Function 3) and scheduling home care visits (Function 4). Elsa's discharge (Function 5) involved a handover to the receiving units (Function 6) under the assumption that she was independently mobile. However, upon arriving home, Elsa faced a staircase that was not considered during her hospital stay (Function 7) or in her transport arrangements (Function 8). This led to a need for her being physically carried upstairs (Function 10) by the care team. The staff had to extend their stay and involve additional personnel and resources.

We're often told about a patient's expected state post‐discharge, only to discover discrepancies upon their arrival. Especially on Fridays, this can leave us without adequate support or mobility aids like a walker or a wheelchair, forcing us to find emergency solutions. (Participant 36, assistant nurse, community care)

### 5.4 Impact from aggregation of system variability

**FIGURE 4:**
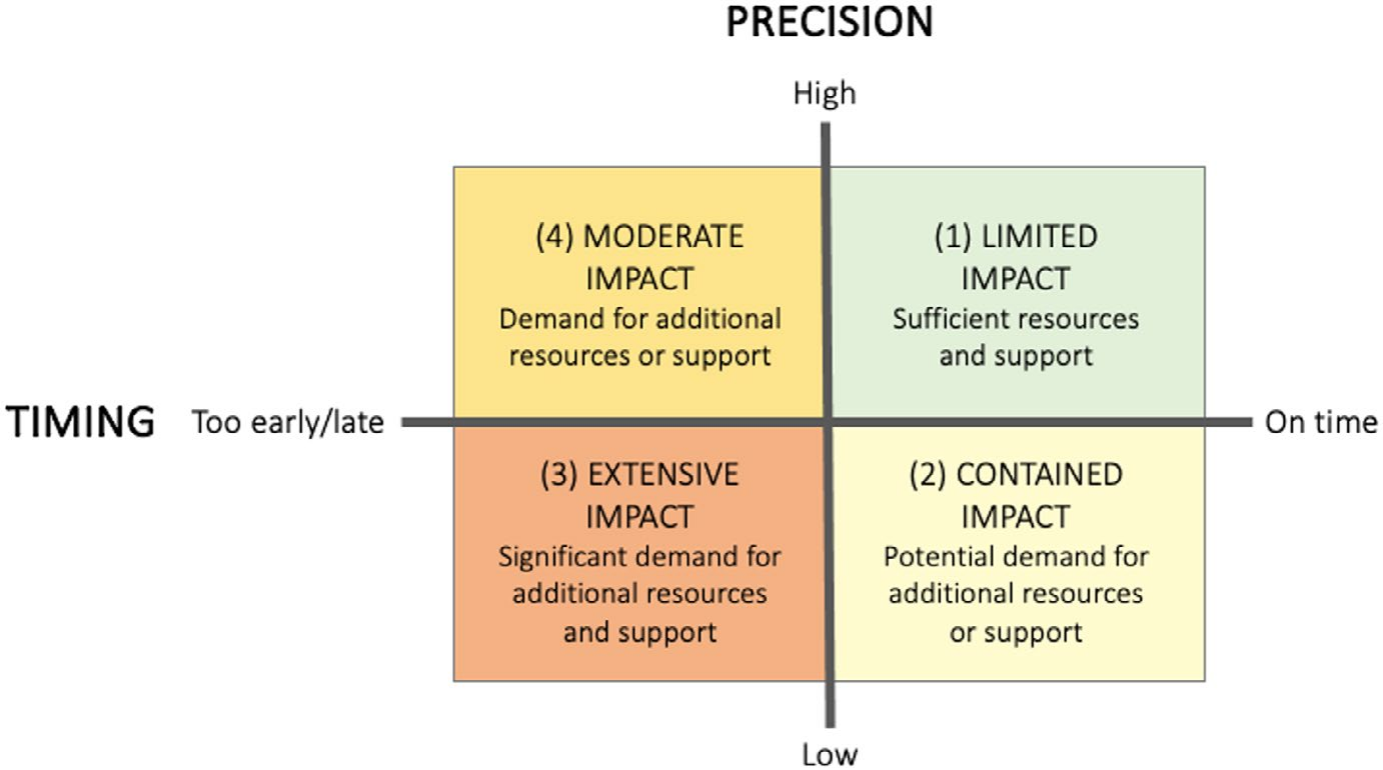
Impact of aggregated system variability in timing and precision within care transitions.

HSCPs face the constant challenge of prioritizing resources effectively when confronted with unanticipated patient needs or logistical complications. System variability, which stems from varying precision in patient assessments and the timing of care transitions, has a significant impact on care delivery. When precision is lacking, essential resources can become overburdened or misallocated, leading to situations where patient needs may be crowded out by other demands, increasing HSCPs' workloads and complicating care delivery. Early discharges, which are often manageable with comprehensive planning, can become problematic when compounded by variability in both timing and precision. For example, patients released late on Fridays without complete information present intricate challenges, especially when resources are already stretched thin. Weekend discharges exacerbate these issues, with regular staff off duty and electronic patient records potentially being incomplete, leaving HSCPs to navigate without clear guidance. Such situations reveal a heightened vulnerability within the system, as the strain on available resources intensifies. The consequences of such system variability are complex, affecting both patients and healthcare organizations, as depicted in Figure 4.

The first quadrant encompasses limited impact scenarios where high precision and optimal timing converge. This creates an efficient care environment with readily available resources, ensuring smooth patient care. In the second quadrant, the impact is contained as low precision is counterbalanced by optimal timing, keeping the system's strain within manageable limits. The third quadrant includes critical situations where low precision coupled with poor timing results in significant demand for resources, straining the system and risking a breakdown of care. The fourth quadrant contains scenarios where issues with timing alone imply moderate impact with a risk for disruptions. Even with maintained precision, inappropriate timing leads to a need for additional efforts and resource reallocation. This model encapsulates the critical balance between timing, precision and adaptability, highlighting the importance of a coordinated approach to maintain care quality and mitigate the need for unplanned resources.

### 5.5 Conditions for safe care transitions and management of system variability

Managing the unpredictability inherent in care transitions is critical for ensuring patient safety. When post‐discharge needs are not accurately anticipated, this can introduce risks and uncertainties. Healthcare professionals indicate that conditions in the home environment are particularly variable and require adaptability in care strategies. The resources planned for a patient's discharge may not always align with actual needs, underscoring the importance of a responsive and flexible healthcare system, along with patient participation. Strategies for managing this variability include ensuring additional resources outside regular hours, creating flexibility in time management, empowering healthcare professionals with autonomy and decision‐making capabilities and improving the discharge planning process with better communication between all parties. For instance, involving the family of a patient and notifying community care about the patient's discharge allow for more efficient scheduling. As an assistant nurse notes, knowing the exact timing of a patient's return home enables the care team to plan more effectively, enhancing the overall care process and workload management:

Our workload peaks in the evening with typically just three of us, as opposed to six during the day. Timely discharges help – for example, knowing [name] will be home by eleven lets us reschedule cleaning tasks to another day to ensure someone is available to meet him. Ideally, if we receive notice a day in advance of a patient's arrival and they return before two in the afternoon, it simplifies the process, whereas later arrivals complicate our evening routine and additional duties. (Participant 11, assistant nurse, community care)

Elsa's scenario underscores the communication gap between hospital care and community care, revealing potential patient safety risks. The unpredictability of home care needs, which are not always apparent during hospitalization, requires anticipatory planning. Community care staff frequently confront unexpected issues following discharge. Although they play a vital role in patient care, they often have limited access to complete patient information and a marginal or non‐existent role in discharge planning. To address patient needs, these key care providers sometimes work longer hours or skip breaks, particularly when unpredictable situations arise, revealing the system's inherent weaknesses. The timing of John's discharge, for example, significantly affected the capacity of HSCPs to tackle sudden challenges. The care provided to John was shaped more by the system's constraints than by John's individual needs. In Elsa's scenario, the community care team could pivot their strategy to better meet her needs.

The ‘safe care transition pathway’, described in Box 3, has been established based on our research as a suggestion of collaborative strategies to enhance adaptability, manage vulnerabilities and reduce risks during transitions of care. This pathway is designed specifically to handle the intricacies and unpredictable nature of discharging patients with complex needs. The aim is to align optimal procedures with the practicalities of everyday work, centring on the well‐being of patients and healthcare professionals alike. The pathway advocates a proactive stance in managing system variability, to promote a smooth and secure discharge process. The recommended actions and strategies are detailed within the pathway, to equip healthcare teams with the capacity to proactively address potential risks and vulnerabilities, thereby promoting safer transitions for patients with complex care needs. Acknowledging and preparing for the potential unpredictability of discharge—especially during riskier times, like Friday evenings or weekends—is vital. When weekend discharges for patients with complex needs are unavoidable, rigorous adherence to the ‘safe care transition pathway’ is essential to ensure continuity and quality of care.

## 6 DISCUSSION

Our research has uncovered significant vulnerabilities in care transitions, underscoring the impact of systemic issues in timing and precision on patient safety, resource allocation and HSCPs' workloads. Notably, outside regular work hours, unforeseen situations reveal the system's fragility, with systemic constraints sometimes prioritized over personalized patient care. As the shift towards home‐based health care continues (OECD, 2023), the findings underscore the urgent need for improved organizational adaptability and robust inter‐professional collaboration.

### 6.1 Organizational adaptability—Addressing vulnerabilities in care transitions

The unpredictability of patient needs in home settings, underscored in this study, makes it abundantly clear that rigid, inflexible organizational structures are incompatible with the evolving healthcare landscape. Organizations must embrace adaptability—not as a virtue, but as a necessity. Resilient health care emphasizes the ability to anticipate, monitor, respond and learn from changing situations (Tong et al., 2008). The patient scenarios of John and Elsa highlight the importance of actualizing these principles, ensuring that care remains responsive and adaptive to patient needs. This is particularly evident in unforeseen situations; the study revealed the fragility of the system and a tendency to prioritize systemic operations over personalized care. Recent studies on resilience in health care have concluded that the healthcare system should be designed around patient needs, not provider convenience (Behrens et al., 2022), which aligns with the principles of person‐centred care.

The scenarios of John and Elsa elucidated how the inherent complexity of the healthcare system introduced unintended gaps in the transition of care—ultimately affecting the individual patient with complex care needs. Healthcare professionals are not the only ones affected by system gaps; patients and families are often forced to bridge such gaps on their own (Vos et al., 2018). Recognizing patients and their families as active partners in the care team is crucial (Eldh et al., 2020; Jerofke‐Owen et al., 2023). Their insights and contributions can significantly enhance the understanding and management of care transitions. Families can mitigate negative impacts by acting as informal caregivers, but their participation should not be taken for granted. In instances where families face their own constraints and cannot provide the necessary support, alternative measures are crucial to maintaining care continuity. The study highlighted that the combined efforts of healthcare professionals, patients and their families are often crucial to upholding the resilience of an overextended healthcare system, with families frequently bearing a significant part of this responsibility.

In scenarios with misalignments between capacity and demand, HSCPs must make tough choices about where to allocate their time and effort. The aggregation of system variability can lead to situations where care providers must navigate these complexities without a safety net, often relying on their judgement and experience to prioritize patient care effectively. These professionals, who work closest to the patients, effectively become the cornerstone for patient safety performance (Bergström & Dekker, 2019; Ekstedt et al., 2022). In the complex adaptive system of health care (Braithwaite et al., 2013), human adaptability is paramount. However, this adaptability can inadvertently conceal system weaknesses, giving an illusion of better performance than what is actually at hand (Raeisi et al., 2019). In the study, the HSCPs modified their work and allocated more time to preserve patient safety, compensating for systemic deficits. Such short‐term adaptations, akin to firefighting, can have unintended and intricate repercussions (Lyng et al., 2021). Previous research applying the lens of resilience suggests that understaffing and limited access to resources may create a self‐reinforcing trap in the system (Wiig et al., 2020).

Our research indicated that system variability can strain resources, posing significant challenges for HSCPs, who operate in an increasingly complex and demanding environment. This raises concerns about the sustainability of current healthcare practices and models. System vulnerabilities that lead to the overburdening of HSCPs are associated with critical issues, including staff burnout, high turnover and decreased capacity to maintain high‐quality care (Salyers et al., 2017). The increasing demands in health care necessitate a re‐evaluation of resource allocation to adequately support professionals. Ensuring that the system can manage growing demands is essential to maintain the quality of patient care.

Resilience in health care goes beyond coping with strains; it involves refining system efficiency to handle challenges effectively (Braithwaite et al., 2015). For assessments to be precise and interventions timely, there is a need for a competent workforce proficient in the specifics of home‐based care. Ongoing training and the provision of competence are essential. A workforce skilled in coordinating and responding to complex care needs is better prepared to anticipate potential pitfalls, and can not only improve the quality of care but also optimize resource utilization. With robust inter‐professional collaboration (Leutz, 2005) and skilled staff, the healthcare system is better equipped to transition towards home‐based care. This approach ensures that patients like John and Elsa receive care that is both effective and efficient.

### 6.2 Learning from patient scenarios—Paving the way for safe care transitions

Building on insights from our prior research (Hedqvist et al., 2023), we would suggest that any delays or inaccuracies in care planning can initiate a domino effect, amplifying risks and potentially jeopardizing patient safety. John's care in particular illustrates the impact of timing and the resulting cascade of events that threatens patient safety. Premature discharge risks insufficient recovery (Coffey et al., 2019), while delays can escalate costs, extend hospitalization or inadvertently introduce hospital‐associated risks (Al‐Yarabi et al., 2022; Bai et al., 2019).

Elsa's story, on the other hand, emphasizes the impact of imprecise or flawed information and the care plans derived from it. Incomplete assessments or insufficient patient‐specific coordination can result in inadequate care strategies, heightened risks and potential harm to the patient (Raeisi et al., 2019). A seamless care transition is especially critical for patients with complex needs. Prior studies have underlined the necessity of establishing and following precise care transition and discharge protocols, with clearly outlined criteria, for such patients (Hedqvist et al., 2023). As a targeted response to these issues, we have developed the ‘safe care transition pathway’, detailed in Box 3. This offers a structured approach to effectively monitoring and managing the inherent variability in care transitions, accommodating the unique needs of patients. It serves as a practical guide for HSCPs managing the transition from hospital to home, enhancing preparedness and predictability for all involved parties, including the home care team, the patient and their family. Central to this pathway is the emphasis on patient participation and family involvement, promoting a mutual relationship where the patient and their family are considered active partners in the care team (Jerofke‐Owen et al., 2023). The pathway directly addresses previous research gaps by providing a holistic strategy that prioritizes patient safety aiming to improve the overall quality of care during care transitions.

### 6.3 Untangling complexity—Is integrated care the answer?

The findings underscored the non‐linear nature of healthcare processes, where seemingly minor variations in patient assessment or care coordination could unpredictably escalate into major challenges. This highlights the inherently unpredictable trajectory of patient care transitions, in line with the characterization of health care as a complex socio‐technical system (Dekker et al., 2011; Rasmussen, 1997). The findings indicated that while there are specific weaknesses in the care transition from hospital to home, these are symptomatic of broader systemic challenges. Addressing them in isolation may provide temporary fixes, but building a truly resilient healthcare system requires a holistic approach. For sustainable change, a holistic review and redesign of the healthcare system are warranted.

Integrated care is a model of care designed to provide coordinated services to individuals with chronic illnesses, covering the entire care spectrum through to a multidisciplinary approach (Danhieux et al., 2021; World Health Organization, 2016). Research has shown that such integration can lead to better patient experiences, improved access to care and higher levels of patient satisfaction (Hughes et al., 2020). However, integration of services introduces its own challenges, as it increases the complexity of connections and dependencies between different healthcare and social service entities (Tran et al., 2021). On the other hand, interdependence can be viewed positively, as it forms the foundation for a mutual relationship that is essential for meaningful participation, involvement and engagement of a patient in their own care (Jerofke‐Owen et al., 2023).

Our research highlighted the imperative of promoting resilience across the healthcare continuum. In an ever‐evolving healthcare landscape, resilience must evolve from a reactive stance into proactive, adaptable strategies that align with the nuanced needs of patients. The findings would suggest that healthcare policymakers, leaders and professionals must look beyond merely identifying systemic weaknesses. The focus should be on developing interventions that not only prioritize patient safety, but also reinforce the resilience and efficiency of the healthcare infrastructure. As the dynamics of health care evolve, confronting such challenges becomes paramount, with an overarching priority on safeguarding patient care. To truly enhance the system, we must consider re‐evaluating existing policies, nurturing a culture centred on inter‐professional collaboration and leveraging technology in ways that meaningfully bridge existing gaps.

Complex problems seldom have simple solutions. This study paints a picture of a healthcare system riddled with challenges, from fragmented care modules to overlapping responsibilities and barriers to effective communication, even when striving for an integrated care approach. Patients and their families face their own vulnerabilities. As they manoeuvre through the complex healthcare maze, they are confronted with risks stemming from potential inconsistencies in their care. This underlines the pressing need for a healthcare system that is truly cohesive and person centred. In essence, the vulnerabilities are twofold: those intrinsic to the healthcare system itself and those affecting patients and their families (Danhieux et al., 2021; O'Hara et al., 2020). Recognizing these vulnerabilities is crucial.

### 6.4 Strengths and limitations of the work

The FRAM has been widely applied in healthcare research, particularly for understanding transitional care and hospital discharges (Buikstra et al., 2020; McGill et al., 2020; O'Hara et al., 2020; Salehi et al., 2021). In this study, the FRAM has been instrumental in visualizing the inter‐ and intra‐organizational complexities of care transitions for patients with complex needs, significantly aiding the data analysis.

This study delves into the complex process of care transitions, involving a wide range of healthcare professionals. Due to the complexity of such transitions, it is difficult to set clear boundaries, as they tend to involve multiple healthcare systems (Greenhalgh & Papoutsi, 2018). Real‐world situations, shaped into narratives and confirmed through member checks, grounded the analysis in clinical reality and enhanced the relevance of the findings. However, it is possible that certain subtleties were not captured fully.

The study's robust data collection encompassed diverse healthcare environments over an extended period, revealing the dynamic nature of inter‐professional collaboration and enriching our understanding. However, there were limitations. The research was based in a distinct area of Sweden's healthcare system, which may restrict the transferability of the findings. Future research should include patient and family experiences to fully grasp the essence of care transitions.

### 6.5 Implications for policy and practice

Policy implications include promoting inter‐professional collaboration and strategic resource allocation, standardizing safe care transition pathways (see example in Box 3), and emphasizing patient participation and family involvement during care transitions. Practical application requires clear communication with patients and families, as well as within and across healthcare teams. Integrating information technology is essential for smooth information flow. Forming collaborative care teams and adhering to patient safety protocols during high‐risk transitions are also crucial steps. Collectively, these measures offer a holistic approach that may alleviate the pressure on families within an overburdened healthcare system and contribute to safer care transitions.

## 7 CONCLUSION

The study illuminated how organizational adaptability impacted patient safety during care transitions for patients with complex care needs. Notably, the repercussions of the identified system gaps were widespread, affecting not only patients and their families, but also healthcare professionals. The study indicated that the current system structure was overly reliant on frontline workers, patients and their families to compensate for systemic shortfalls such as understaffing or limited resource availability. This dependency potentially jeopardizes the sustainability of health care, at both the individual and organizational levels. Clear communication, effective information technology use and collaborative care teams are vital. To improve patient safety, strategies must extend beyond inter‐professional collaboration to include strategic and flexible resource planning, and standardization of safe care transition pathways with patient participation and family involvement. Collectively, these insights provide a roadmap for optimizing care transitions and enhancing patient outcomes. Further work is needed to develop guidance for achieving flexibility in care transitions and contribute to more integrated and adaptive care for patients with complex care needs.

## AUTHOR CONTRIBUTIONS

AH, GP, ME, CL: Made substantial contributions to conception and design or acquisition of data or analysis and interpretation of data. Involved in drafting the manuscript or revising it critically for important intellectual content. Given final approval of the version to be published. Each author should have participated sufficiently in the work to take public responsibility for appropriate portions of the content. Agreed to be accountable for all aspects of the work in ensuring that questions related to the accuracy or integrity of any part of the work are appropriately investigated and resolved.

## FUNDING INFORMATION

The overarching project that this study is part of was funded by the Kamprad Family Foundation for Entrepreneurship, Research and Charity (no: 20190249). The funding body had no input on design, conduct or reporting of the results.

## CONFLICT OF INTEREST STATEMENT

The authors declare no conflicts of interest.

## PEER REVIEW

The peer review history for this article is available at https://www.webofscience.com/api/gateway/wos/peer‐review/10.1111/jan.16203.

## Supporting information


Appendix S1.

Appendix S2.

## Data Availability

The data that support the findings of this study are available on request from the corresponding author. The data are not publicly available due to privacy or ethical restrictions.
