# Supplementary material for: Entangled in complexity: An ethnographic study of organizational adaptability and safe care transitions for patients with complex care needs
Source: J Adv Nurs. 2024 Apr 20;81(9):5528–45. doi: 10.1111/jan.16203 (PMC12371820; doi:10.1111/jan.16203)
Supplement: Supplementary file 1 — Appendix S1. [file JAN-81-5528-s001.docx]

Appendix S2

Functions in the FRAM models illustrating the care transition process

| **Function (i.e., event)** | **Description** | **Identified aspects** | |
| --- | --- | --- | --- |
| Adapt aids in home | The function is concerned with adapting and modifying the patient's home environment to match their evolving needs for aids and equipment. This customization depends on the availability of such tools. The aim is to ensure that the patient can navigate their living space safely and with as much independence as possible. The result is properly installed and functional mobility aids in the patient’s home. This is essential for the safety and independence of the patient, particularly after they have been discharged from hospital. Based on the extent of adaptations needed, the function may take hours to a few days to complete.  The function is primarily performed by community rehabilitation staff. | **Input** | - Altered need of aids |
|  |  | **Output** | - Adequate aids in home |
|  |  | **Preconditions** | - Assessment of the patient's Activities of Daily Living (ADL) and mobility needs - Assessment of home environment regarding compatibility with mobility aids - Patient’s and family’s consent to care efforts and adaptations in home |
|  |  | **Resources** | - Aids available |
|  |  | **Control** | - Patient's and family's preferences |
| Adapt home care | As a patient's condition changes, their home care might need adjustments. These can range from adjustments in medication dosages, incorporation of new therapies, changes in the frequency of visits by healthcare professionals, or the introduction of new care techniques. Continuous monitoring and feedback loops, including regular check-ins with the patient and family, ensure that the care remains effective and satisfactory for the patient. The goal is to enhance quality of life for the patient.  The function is primarily performed by community home care staff such as registered nurses and assistant nurses. | **Input** | - Altered need of home care |
|  |  | **Output** | - Adequate home care efforts |
|  |  | **Preconditions** | - Execution of efforts in home according to plan - Attention to patient health status - Patient’s and family’s consent to care efforts and adaptations in home |
|  |  | **Resources** | - Comprehensive care plan |
|  |  | **Control** | - Protocols and guidelines defining when and how to escalate care |
| Adapt social care in home | As a patient's condition changes, their social care might need adjustments. These can include adjustments in support needed or changes in the frequency of visits by social care staff.  The function is primarily performed by community social services. | **Input** | - Altered need of social care |
|  |  | **Output** | - Adequate social care efforts in home - Staff physically capable of performing heavy lifting |
|  |  | **Preconditions** | - Patient’s and family’s consent to care efforts and adaptations in home |
| Assess ADL and mobility | The ADL and mobility assessment is an evaluation of a patient's capacity for self-care and independent movement. It encompasses assessing ADL such as eating, dressing, and bathing, as well as assessing balance, gait, and the need for mobility aids. The assessment is typically conducted by rehabilitation experts such as physiotherapists or occupational therapists. It informs the level of support and equipment needed after hospital discharge. If specialized rehabilitation staff are unavailable, ward nurses are responsible for this assessment. The insights gained from this process are integral to developing a coordinated care plan that facilitates a patient's seamless transition from hospital to home care.  The function is primarily performed by rehabilitation staff. | **Input** | - Initiated care coordination process |
|  |  | **Output** | - Assessment of the patient's ADL and mobility needs |
|  |  | **Resources** | - Knowledge of patient and family - Assessment of home environment regarding compatibility with mobility aids |
|  |  | **Control** | - Patient's and family's preferences |
| Assess patient in prehospital setting | If there is a perceived need for a prehospital assessment and/or care, the community care staff, family, or patient may call emergency services. The prehospital assessment refers to the medical evaluation and care provided to a patient prior to arrival at hospital, typically conducted by ambulance staff. This evaluation plays a crucial role in identifying the immediate needs of a patient and formulating a preliminary treatment plan.  The function is primarily performed by ambulance staff. | **Input** | - Perceived need of prehospital assessment and care |
|  |  | **Output** | - Need of inpatient care - Emerging needs - Identification of inadequate possibilities of care in home |
|  |  | **Resources** | - Adequate care efforts in home |
|  |  | **Control** | - Medical plan in case of exacerbations - Comprehensive care plan - Agreed-upon plan for care in home - Medical assessment or dialogue |
| Assess patient ready for discharge | Following medical treatment, a patient's health and medical progress are evaluated to determine their readiness for discharge. The attending physician makes a decision on this based on the assessment results. If the patient requires modifications in their home, such as medical equipment, accessibility enhancements, or education for family members, the discharge might be delayed. Furthermore, if there are concerns about the patient’s capabilities due to medical, psychological, or environmental factors, discharge may be postponed until necessary arrangements are finalized.  The function is primarily performed by professionals in in-hospital care, with the attending physician having overarching responsibility. | **Input** | - Decision on medical fitness for discharge |
|  |  | **Output** | - Patient ready for discharge - As soon as possible |
|  |  | **Control** | - Patient’s medical evaluation |
|  |  | **Time** | - Patient nearing end of treatment or hospital stay - Before discharge |
| Carry patient inside | This function is activated as an ad hoc solution under circumstances where the patient cannot enter their home independently and the available mobility aids prove inadequate for assistance. In such situations, additional support or interventions are necessary to ensure the patient's safe entry into their home.  The function is primarily performed by home care staff. | **Input** | - Patient leaves hospital |
|  |  | **Output** | - Patient arrives home |
|  |  | **Resources** | - Staff physically capable of performing heavy lifting |
| Check bed availability* | The attending physician checks if there are beds available on the ward in question. If the patient has a large need for inpatient care and there are no beds available, they might be placed on a different ward. | **Output** | - Availability of beds on ward |
| Clarify housing conditions | This process involves evaluating and ensuring that a patient's home environment is suitable and safe for their recovery or ongoing care needs. It includes thorough collection of information regarding the home's layout, accessibility, and general suitability to support the patient's health and ability to move around.  The function is primarily performed by rehabilitation staff. | **Input** | - Initiated care coordination process |
|  |  | **Output** | - Assessment of home environment regarding compatibility with mobility aids |
|  |  | **Preconditions** | - Patient’s and family’s consent to care efforts and adaptations in home |
|  |  | **Resources** | - Knowledge of patient and family |
| Co-create plan of coordinated care | Collaborative planning involves a multidisciplinary team, including rehabilitation experts, social service officers, physicians, nurses, and other specialists, who work together to formulate a holistic care plan. This plan takes account of the patient's medical needs, preferences, and challenges. After thorough discussions among all parties, a preliminary care plan is co-created to guide the patient's transition and ongoing care.  The function is primarily performed in collaboration between professionals from in-hospital care, primary care, and community care. | **Input** | - Initiated care coordination process |
|  |  | **Output** | - Agreed-upon plan for care in home - Plan for evaluation - Plan for aids in home |
|  |  | **Preconditions** | - Patient's consent for data sharing - Assessment of the patient's ADL and mobility needs |
|  |  | **Resources** | - Knowledge of patient and family - Interprofessional collaboration |
|  |  | **Control** | - Patient's and family's needs - Patient's and family's preferences |
|  |  | **Time** | - During inpatient care |
| Collaborate within and between organizations* | A lot of planning and dialogue takes place within and between the various healthcare organizations, though the patient and family do not appear to be represented. | **Output** | - Interprofessional collaboration |
| Compose medical summary | Upon completion of medical care in the inpatient setting, the attending physician prepares a comprehensive medical summary. This document is not only made accessible digitally through medical record systems, but also provided in printed form for the patient to physically carry home. This summary encompasses critical information such as the patient’s diagnosis, treatments administered, medications prescribed, and specific care instructions to ensure continuity of care post-discharge. It serves as a vital communication tool, serving as a bridge for the transition from hospital to home care, and is instrumental for any subsequent healthcare providers who will be involved in the patient's ongoing treatment and recovery.  The function is primarily performed by the attending physician on the hospital ward. | **Input** | - Patient discharged |
|  |  | **Output** | - Summary of medical care - Medical plan in case of exacerbations - Referral to primary care |
| Consult physician in primary care | When a medical assessment of the patient is necessary, for instance during an exacerbation of a chronic illness, the healthcare team initiates a detailed dialogue with the general practitioner overseeing the patient's care. This consultation aims to evaluate the patient's current health status, discuss any new symptoms, and determine the appropriate course of action. The general practitioner, leveraging their familiarity with the patient's medical history and ongoing health issues, plays a crucial role in adjusting treatment plans, prescribing necessary medications, and/or recommending further interventions.  The function is primarily performed by community nurses, but may also be initiated by ambulance nurses. | **Input** | - Need of medical assessment or dialogue |
|  |  | **Output** | - Decision to re-admit patient - Medical assessment or dialogue |
|  |  | **Resources** | - Agreed-upon plan of care in home |
| Decide if medically fit for discharge | This function involves an assessment of a patient's current health status to determine if they are ready to leave the hospital. Based on the patient's medical evaluation, healthcare professionals make an informed decision, relying on medical records, the latest diagnostic tests, hospital discharge protocols, and the patient's health stability. This process ensures that only patients who have reached a certain health threshold and have been deemed stable are discharged. Feedback from this function might also include recommendations on post-discharge care or a decision to continue inpatient care if the patient is not yet ready for discharge.  The function is primarily performed by the attending physician on the hospital ward. | **Input** | - Patient nearing end of treatment or hospital stay |
|  |  | **Output** | - Decision on medical fitness for discharge - Before discharge |
|  |  | **Control** | - Patient's medical evaluation |
|  |  | **Time** | - During inpatient care |
| Decide upon level of care | An increased need of medical care may require a decision on what would be the most appropriate level of care. To inform this decision, the medical plan and agreed-upon plan for care in home are used. The patient should preferably receive adapted care efforts in their home, but if this is not possible, an escalation of care may be needed, where the patient is admitted to hospital.  The function is primarily performed in collaboration between community care staff, such as registered nurses, in dialogue with the general practitioner overseeing the care of the patient. | **Input** | - Increased need of medical care |
|  |  | **Output** | - Identification of inadequate possibilities of care in home - Need of medical assessment or dialogue - Perceived need of prehospital assessment and care |
|  |  | **Resources** | - Adequate care efforts in home |
|  |  | **Control** | - Medical plan in case of exacerbations - Decision on medical care in home - Comprehensive care plan - Agreed-upon plan for care in home |
| Discharge patient | The attending physician at the hospital ward discharges the patient from inpatient care, thus terminating the inpatient trajectory. The physician assesses the patient's condition and ensures that all care targets are met or will remain post-discharge. Detailed care instructions are provided to the patient, alongside arrangements for follow-up care, ensuring a safe transition to the next phase of recovery.  The function is primarily performed by the attending physician on the hospital ward. | **Input** | - Patient ready for discharge |
|  |  | **Output** | - Patient discharged - Continuous medical plan in primary care - Discharge letter to patient - Updated list of medicines |
|  |  | **Preconditions** | - Patient ready to go home |
|  |  | **Time** | - Agreed-upon time for homecoming |
| Escalate care | When a patient's condition worsens significantly at home, exceeding what community care can manage, hospital readmission may be necessary for more intensive medical care. Such escalation typically occurs when home care efforts are insufficient for a rapidly declining health status or when a severe exacerbation of the patient's illness requires urgent hospital-based medical assessment and treatment. The decision to readmit is made collaboratively between healthcare providers, prioritizing the patient's immediate health needs, and ensuring their access to the appropriate care level.  The function is primarily performed in collaboration between the community home care nurse, ambulance staff, and the general practitioner. | **Input** | - Inadequate possibility of care in home |
|  |  | **Output** | - Decision to re-admit patient - Patient transferred to hospital |
|  |  | **Resources** | - Interprofessional collaboration - Comprehensive care plan |
|  |  | **Control** | - Protocols and guidelines defining when and how to escalate care |
| Handle needs with available resources | Once altered care needs are identified, the immediate challenge lies in effectively addressing these needs within the constraints of the available resources. This involves strategic assessment of existing resources, matching them against care needs, and devising a plan that maximizes patient benefits while ensuring sustainability and efficiency.  The function is primarily performed in collaboration between community care staff, community nurses, and rehabilitation staff. | **Input** | - Emerging needs |
|  |  | **Output** | - Adequate care efforts in home |
|  |  | **Resources** | - Interprofessional collaboration - Comprehensive care plan |
| Identify altered care needs | Identifying altered care needs is a critical aspect of adaptive and person-centered healthcare. As a patient progresses, recovers, or faces new health challenges, their care requirements can change. Through a systematic assessment of the patient's current health status in relation to their previous status, healthcare professionals aim to pinpoint these changes. Such identification ensures that the patient receives the most appropriate and effective care tailored to their evolving needs.  The function is primarily performed in multidisciplinary collaboration. | **Input** | - Monitoring of care efforts - Attention to patient health status - Assessment of efforts in home according to plan |
|  |  | **Output** | - Altered need of social care - Altered need of home care - Altered need of aids - Increased need of medical care - Emerging needs |
|  |  | **Resources** | - Interprofessional collaboration - Comprehensive care plan |
| Inform family of discharge | Involves proactive communication with the patient’s family, typically on the day of discharge, to convey critical information about the patient’s return home. The ward staff makes contact to provide details on the discharge plan, anticipated arrival time, and any specific care requirements or instructions that can aid the patient’s safe and comfortable transition from hospital to home. This helps to ensure that the family can be adequately prepared, and that any necessary adjustments are made in advance to accommodate the patient’s needs. Through dialogue, the family can also address concerns and clarify any uncertainties.  The function is primarily performed by hospital staff, most often a registered nurse. | **Input** | - Patient ready to go home |
|  |  | **Output** | - Family’s understanding and preparedness for the patient’s return - Agreed-upon time for homecoming |
|  |  | **Time** | - Before discharge |
| Install aids in home | This function is carried out by rehabilitation staff who visit the patient at home post-discharge to install and ensure the proper functioning of necessary aids (e.g., grab bars, ramps, stairlifts, walking aids). The process includes testing aids for suitability and teaching the patient how to use them effectively. Installation times can vary, and in some cases, patients may have to wait several days before they can fully utilize certain aids or adaptations.  The function is primarily performed by community rehabilitation staff. | **Input** | - Patient ready for discharge |
|  |  | **Output** | - Aids in home |
|  |  | **Preconditions** | - Assessment of the patient's ADL and mobility needs - Aids available |
|  |  | **Control** | - Plan for aids in home |
|  |  | **Time** | - Patient leaves hospital |
| Involve patient and family* | This function entails healthcare professionals actively involving a patient and their family in making informed decisions about the patient’s care. It includes discussing care plans, treatment options, and expected outcomes, ensuring that the patient's and family's preferences, perspectives, and consent are clearly understood and considered. The aim is to foster shared decision-making, align care with individual needs and values, and ensure that patients and families are integral partners in the healthcare process. | **Output** | - Knowledge of patient and family - Patient’s and family’s preferences - Patient's and family's needs - Patient’s and family’s consent to care efforts and adaptations in home |
| Monitor and evaluate care efforts in home | Entails a thorough assessment of the care activities planned and executed in the patient’s home. This evaluation is conducted collaboratively within a few days post-discharge, involving the patient, their family members, and healthcare professionals. This collective review process identifies aspects of the care that have been successful as well as areas needing improvement. Adjustments are made accordingly, ensuring that the care efforts are responsive to the patient’s needs and optimized for efficacy.  The function is primarily performed in collaboration between the community team of registered nurses, assistant nurses, and rehabilitation staff. | **Input** | - Patient arrives home |
|  |  | **Output** | - Monitoring of care efforts - Assessment of efforts in home according to plan |
|  |  | **Preconditions** | - Execution of efforts in home according to plan |
|  |  | **Control** | - Agreed-upon plan for care in home - Comprehensive care plan - Plan for evaluation |
|  |  | **Time** | - Days after homecoming |
| Notify receiving units of pending discharge | This step prepares receiving units for a patient’s discharge. It entails promptly notifying care providers about the patient’s pending transition, allowing them to prepare adequately for the patient’s arrival. This can help to ensure a seamless continuation of care, with necessary preparations made to avoid any treatment gaps. The receiving unit is thus able to mobilize resources and staff, and plan for any required interventions, enhancing patient safety and experiences, while also reducing the likelihood of readmission.  The function is primarily performed by the care coordinator at the hospital ward. | **Input** | - Patient ready for discharge |
|  |  | **Output** | - Units informed about discharge |
|  |  | **Time** | - As soon as possible |
| Obtain consent for data sharing* | Involves securing documented approval from the patient, ensuring that all legal requirements are met and the patient is fully informed about what data sharing entails. This step, carried out prior to any data sharing, requires the use of consent forms and the involvement of trained staff. The successful outcome of this function is authorized sharing of patient data, based on consent. This function is time-bound and needs to be completed before any data sharing can legally and ethically occur. | **Output** | - Patient's consent for data sharing |
| Order transport | This involves organizing the appropriate mode of transportation for a patient post-discharge. With account taken of the patient's health status, physical mobility, and home location, professionals at the hospital coordinate internal or external transportation services to ensure a safe journey home, involving either the patient’s family or transportation services.  The function is primarily performed by assistant nurses or nurses on the hospital ward. | **Input** | - Patient discharged |
|  |  | **Output** | - Patient leaves hospital - Patient arrives home |
|  |  | **Preconditions** | - Patient ready to go home - Aids in home |
|  |  | **Control** | - Assessment of the patient's ADL and mobility needs |
| Perform care transition | The care transition from hospital to home takes place in connection with the patient being discharged by the attending physician. Healthcare professionals on the ward ensure that the patient is returned home to their accommodations and any necessary transportation is ordered.  The function is primarily performed by hospital staff. | **Input** | - Patient discharged |
|  |  | **Output** | - Patient leaves hospital - Days after homecoming |
|  |  | **Preconditions** | - Updated list of medicines - Medicines ready for homecoming - Prepared community home care efforts |
|  |  | **Resources** | - Information from inpatient care - Continuous medical plan in primary care - Discharge letter to patient |
|  |  | **Control** | - Patient ready to go home - Family's understanding and preparedness for the patient's return |
| Perform coordinated care in home | Self-care and care efforts are carried out in the home by the patient, family, rehabilitation staff and social care staff along with registered nurses from the community. The intended care is based on the medical plan from primary care and the agreed-upon plan devised during the care planning before discharge. When returning home from hospital, the patient receives information both verbally and in writing from the hospital regarding their care and treatment, including any medicine changes.  The function is primarily performed in collaboration by community staff. | **Input** | - Patient arrives home |
|  |  | **Output** | - Execution of efforts in home according to plan - Attention to patient health status |
|  |  | **Preconditions** | - Medical plan and treatment in primary care - Adequate home care efforts - Adequate aids in home - Adequate social care efforts in home - Adequate care efforts in home - Patient’s and family’s consent to care efforts and adaptations in home - Updated list of medicines - Medicines ready for homecoming - Patient received at home |
|  |  | **Resources** | - Information from inpatient care - Booked social care staff - Aids in home - Summary of medical care |
|  |  | **Control** | - Agreed-upon plan for care in home - Comprehensive care plan - Decision on medical care in home |
| Plan and order aids | The function centers on determining the patient's mobility requirements following a comprehensive assessment. By collaborating with rehabilitation staff and considering both medical needs and the specifics of the patient's living environment, the most appropriate mobility aids can be identified. These aids aim to ensure safe and comfortable movement for the patient, both indoors and outside. Beyond medical criteria, patient preferences play a crucial role in the selection process, aiming to bolster the patient's autonomy and well-being. Based on the assessment of the patient's ADL and mobility, rehabilitation professionals outline the necessary aids, subsequently ordering them to guarantee their availability upon the patient's return home.  The function is primarily performed by rehabilitation staff. | **Input** | - Patient nearing end of treatment or hospital stay |
|  |  | **Output** | - Aids available |
|  |  | **Preconditions** | - Assessment of the patient's ADL and mobility needs - Agreed-upon plan for care in home - Assessment of home environment regarding compatibility with mobility aids |
| Plan and schedule social care staff | Upon deciding the patient's discharge date, the care coordinator arranges home visits and schedules social care staff accordingly. They inform the care team about the patient's return and necessary interventions. Timely planning increases the likelihood of adequately staffing these visits alongside pre-existing duties.  The function is primarily performed by social care staff along with their coordinators and managers. | **Input** | - Units informed of discharge |
|  |  | **Output** | - Booked social care staff - Informed social care staff |
|  |  | **Preconditions** | - Agreed-upon plan for care in home |
|  |  | **Control** | - Agreed-upon time for homecoming |
| Plan home care | Community home care nurses facilitate the patient's seamless transition back home by addressing key care elements. They handle wound care, prepare supplies, and liaise with specialists as required. Medication management involves reviewing prescriptions and ensuring accurate dosages. They also coordinate the delivery and setup of medical equipment and provide essential training. Nurses educate patients and their families for confident home care. A proactive monitoring strategy with follow-up visits is established to refine care as needed. Collaboration with other healthcare professionals solidifies a comprehensive approach to patient well-being.  The function is primarily performed by care coordinators and registered nurses in the community home care. | **Input** | - Patient ready for discharge |
|  |  | **Output** | - Comprehensive care plan - Prepared community home care efforts - Protocols and guidelines defining when and how to escalate care |
|  |  | **Preconditions** | - Agreed-upon plan for care in home |
|  |  | **Resources** | - Interprofessional collaboration - Information from inpatient care - Patient's and family's preferences |
| Prepare care transition to home | The process of preparing for a patient's discharge involves multiple steps to ensure a smooth transition from hospital to home. It begins with a discharge planning meeting where the patient and their family engage in discussions with healthcare professionals from both discharging and receiving care units. During this meeting, necessary aids are identified, and arrangements are made for their provision and installation in the patient's home. Social care staff are informed about the patient's expected arrival time at home and the specific care measures required. Registered nurses in home care then develop comprehensive care plans and medical records for the patient. They also schedule home visits, manage medication administration, and organize home care efforts to be implemented upon the patient's return. The function is performed on the day of discharge, at the latest.  The function is primarily performed in collaboration between care coordinators at hospital and the community care team. | **Input** | - Patient ready for discharge - Units informed of discharge |
|  |  | **Output** | - Patient ready to go home - Community care efforts ready - Before discharge - Medicines ready for homecoming |
|  |  | **Preconditions** | - Patient’s and family’s consent to care efforts and adaptations in home - Family's understanding and preparedness for the patient's return |
|  |  | **Resources** | - Plan regarding aids in home - Informed social care staff |
|  |  | **Control** | - Agreed-upon plan for care in home - Units informed of discharge - Patient's and family's preferences |
| Re-admit patient | This step involves the procedures and evaluations necessary when a patient requires readmission to the hospital after discharge. Triggered by complications, emergent symptoms, or worsening medical conditions, this function guarantees rapid assessment and provision of appropriate care. The decision-making process is informed by the patient's medical records and current health status, directing the treatment and care approach upon readmission.  The function is primarily performed by the attending physician on the hospital ward. | **Input** | - Patient transferred to hospital |
|  |  | **Output** | - During inpatient care |
|  |  | **Preconditions** | - Need of inpatient care |
|  |  | **Resources** | - Availability of beds on ward |
|  |  | **Control** | - Decision to re-admit patient |
| Receive patient in home | Upon patient discharge, home care or rehabilitation staff are ready to receive the patient in their home. This entails healthcare professionals being scheduled, informed about and prepared for the patient’s needs. Community care ensures that necessary resources, like medical equipment, are available. Clear communication with social care staff is vital for understanding patient needs. Care is customized based on the patient’s medical history, current status, and emotional well-being. Safety is prioritized, with instant support in mobility, medication, and wound care, ensuring a safe and supportive patient environment.  The function is primarily performed by the community team, often home care staff or rehabilitation staff. | **Input** | - Patient arrives home |
|  |  | **Output** | - Patient received in home |
|  |  | **Preconditions** | - Community care efforts ready - Booked social care staff - Informed social care staff - Aids in home |
|  |  | **Resources** | - Information from inpatient care |
|  |  | **Control** | - Medical plan in case of exacerbations |
|  |  | **Time** | - Agreed-upon time for homecoming |
| Resume medical responsibility in primary care | The general practitioner in primary care receives a referral from the attending physician at the hospital, providing guidelines for post-hospitalization follow-up or additional medical actions. This referral can also outline aspects of home-based care.  The function is primarily performed by general practitioners in primary care. | **Input** | - Referral to primary care |
|  |  | **Output** | - Medical plan and treatment in primary care - Decision on medical care in home |
|  |  | **Preconditions** | - Summary of medical care |
|  |  | **Resources** | - Medical plan in case of exacerbations |
| Partake of information from other caregivers* | Physicians at the hospital can access medical records or liaise with primary care to review a patient’s long-term care plans. Data sharing requires the patient’s consent. | **Output** | - Documentation from primary care |
|  |  | **Preconditions** | - Patient’s consent for data sharing |
| Transfer information from inpatient care | This pertains to the secure and efficient transfer of a patient’s data from inpatient settings to other healthcare professionals or departments, and to the patient and family, facilitating continuity of care. This could be vital when a patient is being discharged, transitioning to outpatient care, or being referred to specialists. The process is crucial in ensuring that subsequent care providers have all the information necessary to provide optimal treatment, and it plays a pivotal role in preventing gaps or errors in care. The information is provided both verbally and in writing on the day of discharge, at the latest.  The function is primarily performed by hospital staff such as registered nurses and physicians. | **Input** | - Patient ready to go home |
|  |  | **Output** | - Information from inpatient care |
|  |  | **Preconditions** | - Patient’s consent for data sharing |
| Transport patient to hospital* | The primary goal is to stabilize and safely transport the patient to hospital, providing essential care during transport if necessary, and ensure a seamless handover to hospital staff upon arrival. | **Output** | - Patient transferred to hospital |
| Treat patient in hospital* | The hospital ward's attending physician oversees and administers treatment to the patient. The physician may consult the patient's medical records, review care provided in primary care, and liaise with the patient's general practitioner to discuss ongoing care, treatment, and future planning. | **Output** | - Patient nearing end of treatment or hospital stay - Patient's medical evaluation - During inpatient care |
|  |  | **Resources** | - Documentation from primary care - Interprofessional collaboration |

* Background function; a supporting process that indirectly influences the primary activities within a system, ensuring the main functions can operate effectively.
