# Supplementary material for: Entangled in complexity: An ethnographic study of organizational adaptability and safe care transitions for patients with complex care needs
Source: J Adv Nurs. 2024 Apr 20;81(9):5528–45. doi: 10.1111/jan.16203 (PMC12371820; doi:10.1111/jan.16203)
Supplement: Supplementary file 2 — Appendix S2. [file JAN-81-5528-s002.docx]

Entangled in complexity: An ethnographic study of organizational adaptability and safe care transitions for patients with complex care needs

Consolidated criteria for reporting qualitative studies (COREQ): 32-item checklist

Developed from:

Tong A, Sainsbury P, Craig J. Consolidated criteria for reporting qualitative research (COREQ): a

32 item checklist for interviews and focus groups. International Journal for Quality in Health Care.

2007. Volume 19, Number 6: pp. 349 – 357

| No Item | Guide question / description | Reported on page # |
| --- | --- | --- |
| **Domain 1: Research team**  **and reflexivity** |  |  |
| *Personal Characteristics* |  |  |
| 1. Interviewer/ Facilitator | Which author/s conducted the interview or focus group? | Performed by the first author.  See Methods, Data collection  P.13-15 and author contributions |
| 1. Credentials | What were the researcher’s credentials? E.g. PhD, MD | The researchers were three PhDs, and one PhD student.  See Title page |
| 1. Occupation | What was their occupation at the time of the study? | The researchers were three PhDs and one PhD student, working at the ambulance service. See Title page (affiliations) |
| 1. Gender | Was the researcher male or female? | Female |
| 1. Experience and training | What experience or training did the researcher have? | The first author is a PhD student with supervision by the co-authors who had experience in this method.  Potential author bias was handled by reflexive discussion and peer debriefing during the analysis process.  See Trustworthiness and reflexivity p.21 |
| *Relationship with participants* |  |  |
| 1. Relationship established | Was a relationship established prior to study commencement? | Researchers established contact with top managers and heads of departments. Informants did not have any relationship with the researcher prior to the study.  See Method, Recruitment of participants p.12 |
| 1. Participant knowledge of the interviewer | What did the participants know about the researcher? e.g. personal goals, reasons for doing the research | Informants were introduced to the research by the heads of departments and did not have contact with the researchers prior to interviews or observations. During the observations, the researcher was present and recognizable to the participants, but not an active participant with a role in the social context. See Methods, Participant observations p.14 |
| 1. Interviewer characteristics | What characteristics were reported about the interviewer/facilitator? e.g. Bias, assumptions, reasons and interests in the research topic | Basic information was reported about the interviewer for the participant to get a picture of the person to meet with. |
| **Domain 2: study design** |  |  |
| *Theoretical framework* |  |  |
| 1. Methodological orientation and Theory | What methodological orientation was stated to underpin the study? e.g. grounded theory,  discourse analysis, ethnography, phenomenology, content analysis | Ethnography  See Methods, Design p.9 |
| *Participant selection* |  |  |
| 1. Sampling | How were participants selected? e.g. purposive, convenience, consecutive, snowball | A purposive sampling approach was used.  See Methods, Recruitment of participants p.12 |
| 1. Method of approach | How were participants approached? e.g. face-to-face, telephone, mail, email | See Methods, Recruitment of participants p.12 |
| 1. Sample size | How many participants were in the study? | In total, 37 participants.  See Methods, data collection p.13 and Table 1 p.16 |
| 1. Non-participation | How many people refused to participate or dropped out? Reasons? | None |
| *Setting* |  |  |
| 1. Setting of data collection | Where was the data collected? e.g. home, clinic, workplace | All observations were executed at clinic/workplace while the setting for the interviews was chosen by convenience of the participant and was thus performed via telephone or skype/zoom from home or workplace.  See Methods, Data collection p.13-15 |
| 1. Presence of non-participants | Was anyone else present besides the participants and researchers? | During the formal interviews, only the interviewer and the interviewee were present. During observations in the clinical setting, there were at times also patients and next of kin present, however they were not observed  See Methods, Data collection p.13-15 |
| 1. Description of sample | What are the important characteristics of the sample? e.g. demographic data, date | The importance of the sample is the cross-organizational representation and diversity of healthcare professionals which is reported in Methods, Recruitment of participants p.13 and Table 1 p.16 |
| *Data collection* |  |  |
| 1. Interview guide | Were questions, prompts, guides provided by the authors? Was it pilot tested? | An interview guide with open-ended questions were created and used. Interview techniques featured open-ended questions and active listening, encouraging candid participant narratives, and capturing nuanced experiences.  See Methods, Data collection p.15 and Trustworthiness and reflexivity p.21 |
| 1. Repeat interviews | Were repeat interviews carried out? If yes, how many? | No |
| 1. Audio/visual recording | Did the research use audio or visual recording to collect the data? | Yes the interviews were audio recorded, however not the observations.  See Methods, Data collection p.14-15 |
| 1. Field notes | Were field notes made during and/or after the interview or focus group? | Field notes were written adjacent to observations.  See Methods, Data collection p.14-15 |
| 1. Duration | What was the duration of the interviews or focus group? | The informal interviews could range from between just a few minutes to up to an hour, while the formal interviews on average lasted about 45 minutes. The observations were limited to no more than three consecutive hours to avoid observer fatigue.  See Methods, Data collection, Participant observations and Interviews p.14-15 |
| 1. Data saturation | Was data saturation discussed? | No, but data triangulation, member checking and peer debriefing were used to enhance the rigor and validity of this study,  See Trustworthiness and reflexivity p.21 |
| 1. Transcripts returned | Were transcripts returned to participants for comment and/or correction? | The draft models from the analysis were sense checked with participants. |
| **Domain 3: analysis and findings** |  |  |
| *Data analysis* |  |  |
| 1. Number of data coders | How many data coders coded the data? | The main author coded the data while the co-authors contributed to validation of coding  See author contributions.  See Methods, Data analysis p.17-20 |
| 1. Description of the coding tree | Did authors provide a description of the coding tree? | In part. The coding resulted in functions of the FRAM model with detailed descriptions of each function.  See Methods, Data analysis p.17-19 |
| 1. Derivation of themes | Were themes identified in advance or derived from the data? | Derived from data as the analysis was inductive  See Methods, Data analysis p.17-20 |
| 1. Software | What software, if applicable, was used to manage the data? | Microsoft Excel |
| 1. Participant checking | Did participants provide feedback on the findings? | Author biases were handled by peer debriefing and participant confirmation of the findings as the authors reiterated and confirmed emerging findings with healthcare and social care professionals outside the study. |
| *Reporting* |  |  |
| 1. Quotations presented | Were participant quotations presented to illustrate the themes / findings? Was each quotation identified? e.g. participant number? | Yes. Each quotation was identified with participant´s number and profession.  See Result section – for example p.27 |
| 1. Data and findings consistent | Was there consistency between the data presented and the findings? | Consistency between data and the findings is presented with quotations and in the patient scenarios. |
| 1. Clarity of major themes | Were major themes clearly presented in the findings? | Yes, through the themes of timing and precision in the patient scenarios, along with the major themes in the pathway (box).  See Results section p.26-34 and box 3 |
| 1. Clarity of minor themes | Is there a description of diverse cases or discussion of minor themes? | Yes, as evident in the FRAM models and the comparison on potential and actual variability. Furthermore, minor themes are presented in the pathway (box 3).  See Results section p.21-26 and Box 3 |
